# Factors Associated with Loneliness Among Older Adults Who Experienced Homelessness: Results from the HOPE HOME Study

**DOI:** 10.1007/s11606-025-09818-7

**Published:** 2025-09-10

**Authors:** Yeqing Yuan, Jennifer L. Evans, Torsten B. Neilands, Karen Valle, Cheyenne M. Garcia, Margot B. Kushel

**Affiliations:** 1 UCSF Benioff Homelessness and Housing Initiative, University of California, San Francisco, CA USA; 2 UCSF Division of Health Equity and Society, University of California, San Francisco, CA USA; 3 School of Social Work, Simmons University, Boston, MA USA; 4 UCSF Division of Prevention Science, Department of Medicine, University of California, San Francisco, CA USA

**Keywords:** homelessness, housing, loneliness, older adults

## Abstract

**Background:**

Older homeless-experienced adults are at higher risk of loneliness than general older adults. Loneliness is associated with multiple adverse health and mental health outcomes. Less is known about factors contributing to loneliness among older adults who experience homelessness. This study aims to examine the relationship between relevant factors and loneliness.

**Methods:**

We used data collected from January 2015 to February 2023 from the Health Outcomes in People Experiencing Homelessness in Older Middle agE (HOPE HOME) study, an ongoing longitudinal cohort study of older adults, homeless at study entry, conducted in Oakland, CA. We used structural equation modeling (SEM) to investigate the relationships between trauma, mental health status, physical health and functional status, housing factors, social support, and substance use and loneliness.

**Results:**

Our analytic sample consisted of 385 participants who completed 2099 assessments. The majority of participants were men (74.8%), Black (82.1%), and not partnered (87.5%). At the first loneliness interview, 41.8% remained homeless, and 39.2% met the criteria for loneliness. Poor mental health and a lack of social support were associated with loneliness. Poor physical health and functional status had an indirect effect on increased loneliness through worsening mental health status. Experiences of trauma had an indirect effect on increased loneliness through worsening mental health status. The Shelter-In-Place mandate was associated with decreased loneliness. We found no association between regaining housing and decreased loneliness.

**Conclusion:**

In a longitudinal study of older adults, homeless at study entry, we found that mental health had direct impacts on loneliness, and other factors (i.e., trauma, physical health, and functional status) contributed to increased loneliness through their negative effects on mental health. Regaining housing was not associated with a decrease in loneliness. These findings highlight the need for interventions beyond housing, including mental healthcare, trauma-informed support, and social engagement opportunities.

## INTRODUCTION

Approximately a quarter of adults aged 70 and older in the USA experience loneliness.^1^ Loneliness, different from the objective state of social isolation, is the subjective feeling of distress from a perceived gap between desired and actual social contact.^2,3^ Older adults who experience loneliness are at greater risk of adverse physical health and mental health outcomes, including worsening chronic conditions (e.g., diabetes and cardiovascular disease), cognitive impairment, depression, accelerated aging, disability, and mortality.^4^ Loneliness is associated with a higher frequency of healthcare visits.^5^

In the general older adult populations, sociodemographic characteristics (e.g., older age, not being married/partnered), physical health problems (e.g., chronic health conditions), mental health problems (e.g., depression),^6–9^ and social factors (e.g., a limited social network) are associated with loneliness.^9–11^

Individuals who experience homelessness are at a higher risk for loneliness,^12^ but there is less research on the factors associated with loneliness in older homeless adults. Homeless adults are considered “older” by age 50 because of their high prevalence of geriatric conditions and premature mortality.^13,14^ Approximately half of the single homeless populations is aged 50 and older; this proportion has increased since the early 1990 s.^15,16^ The proportion of homeless adults 65 and older is expected to triple between 2017 and 2030.^17^ Our research found that 40% of older (age 50 and older) homeless-experienced adults (those with current or recent experience of homelessness) experience loneliness, more than twice the prevalence in the general older adults population.^10,18,19^ Compared to housed adults, homeless adults report more adverse childhood experiences and trauma, less community connectedness, and more social isolation.^12,20^ Homeless older adults have a higher burden of mental health and substance use problems, which may be exacerbated by social isolation and co-occurring geriatric conditions.^21–23^ All of these may contribute to loneliness.

Little is known about how these individual risk factors interact and influence one another to shape the experience of loneliness in this population.^24,25^ Understanding the mechanisms of loneliness will inform interventions to address it. In a longitudinal cohort of older adults who were experiencing homelessness when enrolled, we used structural equation modeling (SEM) to investigate relationships between relevant factors and loneliness. We hypothesized that declining physical health and functional status, lower social support, worse mental health, increased substance use, and less stable housing would be associated with increased loneliness. Additionally, we hypothesized that poor physical health and functional impairments would be associated with worse mental health, and that trauma exposure would negatively impact mental health status. Finally, we hypothesized that poor physical health and functional status and trauma exposure would contribute to loneliness indirectly through their negative impact on mental health.

## METHODS

### Study Design

The Health Outcomes in People Experiencing Homelessness in Older Middle agE (HOPE HOME) study is a cohort study of health and life course events among older adults experiencing homelessness in Oakland, CA. We used purposive venue-based sampling to recruit 450 participants aged 50 and older in 2013–2014 (*n* = 350; HOPE HOME 1) and 53 and older in 2017–2018 (*n* = 100; HOPE HOME 2).^26,27^ The Institutional Review Board at the University of California, San Francisco, approved the study.

### Study Participants

Participants were eligible for the study if they were:

1. Homeless at study enrollment as defined by the Homeless Emergency Assistance and Rapid Transition to Housing (HEARTH) Act,^28^;
2. English-speaking;
3. Age 50 or 53 and older for HOPE HOME 1 and HOPE HOME 2, respectively; and
4. Able to provide informed consent using a teach-back method.^29^

Trained staff interviewed participants at baseline and every 6 months, checking in monthly to enhance follow-up. We provided participants with incentives of $15 ($20 starting 2017) for enrollment and follow-up interviews and $5 for monthly check-ins. We included participants active in the study in 2015 when loneliness assessment began. We analyzed data through February 2023.

## MEASURES

### Demographics

We collected demographic information, including age, sex, gender identity, marital status (partnered vs. not partnered), and educational level. We dichotomized education level as less than a high school diploma, or high school diploma/GED and higher. Due to the impacts of structural racism on loneliness, we included a measure of race/ethnicity (Black, white, Latino/a, other). We adjusted the model for the following demographic factors: age, sex, gender identity, and race/ethnicity. Due to small cell size, we collapsed race/ethnicity to Black/non-Black in models.

### Dependent Variable: Loneliness

We assessed loneliness every 6 months using the validated UCLA 3-item scale,^30^ starting in 2015 with HOPE HOME participants and including those later enrolled in the HOPE HOME 2 subsample. We summed scores for each question to obtain the final loneliness score (range, 3–9; a score of ≥ 6 indicating loneliness).^31^

### Independent Variables

#### Time-Constant Variables

##### Childhood Trauma

We assessed childhood trauma as any emotional, physical, or sexual violence before age 18.

#### Time-varying variables

##### Substance Use

We assessed substance use (non-prescribed opioids, cocaine, and amphetamines) in the prior 6 months using the World Health Organization Alcohol, Smoking, and Substance Involvement Screening Test (ASSIST; scores ≥ 4 indicating moderate to severe substance use (versus no or mild use)).^32^ We assessed binge drinking of alcohol by asking participants whether they had more than six drinks in one sitting at least monthly.^33^

##### Mental Health

We defined moderate-to-severe depressive symptoms as a score of ≥ 22 on the Center for Epidemiologic Studies Depression Scale (CES-D).^34^ Consistent with previous literature, we considered a score of ≥ 3 on the Primary Care Post-Traumatic Stress Disorder (PTSD) Screen to indicate PTSD.^35^ We assessed anxiety, hallucinations, cognitive difficulty, and suicidal ideation in the past 6 months using modified questions from the Addiction Severity Index (ASI).^36^

##### Physical Health and Functional Status

To assess chronic health conditions, we used National Health Interview Survey (NHIS) questions, asking participants if a healthcare provider had diagnosed them with hypertension, lung disease (COPD or asthma), coronary artery disease or congestive heart failure, arthritis, or liver disease (including hepatitis and or cirrhosis).^37^ We dichotomized the number of chronic diseases as 0–1 or 2 or more. We assessed urinary incontinence by asking participants how often they leaked urine in the past month and dichotomized the number as none versus any.^38^ To assess functional status, we asked participants if they had difficulty performing any of the five activities of daily living (ADLs).^39^ We measured if participants had difficulty performing any of the six instrumental activities of daily living (IADLs) using the Brief Instrumental Functioning Scale (BIFS), which has been validated in homeless populations.^40^ We defined ADL and iADL impairment as difficulty performing at least one activity on each respective measure. We asked if participants have trouble seeing, even with glasses if needed, if they have any hearing problems if they experienced any pain or took pain medication in the past week, and if they experienced any falls in the past 6 months.^41^

##### Social Support

We structured our social support variables based on the conceptualization that it comprises emotional, instrumental, and informational components.^42^ We assessed emotional support by asking how many close friends or family members participants could confide in about themselves and their problems (0, 1–5, or 6 or more confidants).^43,44^ We assessed instrumental support using the 8-item version of the Patient-Reported Outcomes Measurement Information System (PROMIS) Instrumental Support scale.^45^ Consistent with literature that includes healthcare providers as a source of informational support,^46^ we assessed whether participants had a regular healthcare provider, defined as a non-emergency department source of care. We included marital status, categorized as partnered versus not partnered, as significant others can provide emotional, instrumental, or informational support depending on the relationship and context.^47^

##### Housing Stability

We categorized housing status at the time of the interview in three ways: homeless (according to HEARTH criteria), housed (not meeting HEARTH criteria), or living in a skilled nursing facility. Participants reported the cumulative number of years experiencing homelessness at their first interview. We assessed the number of nights spent unsheltered and in emergency shelters in the past 6 months. Participants reported whether they had received a housing subsidy or voucher or resided in permanent supportive housing (PSH) in the past 6 months.

##### Recent Interpersonal Trauma

We assessed recent interpersonal trauma by asking participants whether they had experienced verbal, physical, or sexual abuse in the last 6 months.

##### Impact of COVID-19

To assess the effect of the COVID-19 Shelter-In-Place (SIP) mandate in effect from March 18, 2020–June 15, 2021, we created an indicator variable coded yes/no based on the visit date and evaluated the effect on loneliness.

## STATISTICAL ANALYSIS

**Figure 1:**
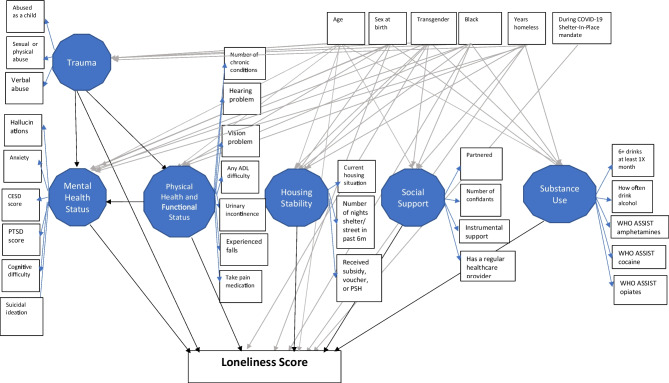
Conceptual model.

After examining descriptive statistics and confirming missing data below 5%, we developed an initial SEM model based on the HOPE HOME dataset literature, theory, and available measures. The initial model contains six direct paths linking latent factors to loneliness (substance use, social support, housing stability, physical health, and functional status, mental health status, and trauma) and three indirect paths between:

1. Experience of trauma to loneliness via mental health;
2. Experience of trauma to loneliness via physical and functional status; and
3. Physical and functional status to loneliness via mental health (Fig. 1).

We refined and refit the model after eliminating one non-significant direct path between substance use and loneliness (Fig. 3). We fit SEM models with the unweighted least squares mean and variance-adjusted estimator (ULSMV) for categorical data. We adjusted standard errors to cluster repeated observations from participants with the TYPE = COMPLEX option. To account for asymmetric distributions of the indirect effects, we report cluster-adjusted bootstrap-based confidence intervals with 5000 requested replicate samples for direct, indirect, and total effects. We considered the effect significant at *p* < 0.05 if the bootstrap-based 95% CI excluded zero.^48^

We assessed the global exact fit for each model using the ULSMV estimated chi-square, and because the chi-square statistic may be sensitive to trivial departures from perfect model-data fit, we used the following approximate fit indices: root mean square error of approximation (RMSEA), standardized root mean square residual (SRMR), comparative fit index (CFI).^49^ We report unstandardized estimate (B) and 95% confidence intervals (Table 2) as well as the standardized parameter estimate (*B*) (Fig. 3).

We computed descriptive statistics for the study population using Stata version 17^50^ and fit structural equation models using M*Plus* version 8.11.^51^

## RESULTS

**Figure 2:**
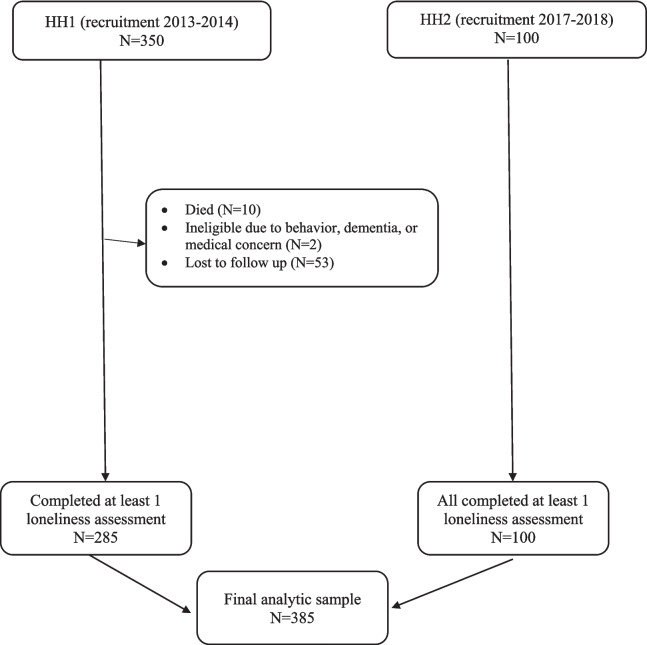
Study flowchart

We analyzed data from 385 participants who remained in the study in 2015 and completed at least one loneliness assessment after enrolling in HOPE HOME 1 (2013–2014) or HOPE HOME 2 (2017–2018). These participants contributed a total of 2099 interviews (median assessments, 9; range, 1–12) (Fig. 2). The majority were men (73.8%), Black (82.1%), and not partnered (87.5%) (Table 1). At baseline, participants reported a median of 3 years of homelessness; 41.8% were homeless at the time of their first loneliness interview, and 39.2% met the criteria for loneliness.

**Table 1:** Characteristics of the Health Outcomes in People Experiencing Homelessness in Older Middle Age (HOPE HOME) Study Participants, Oakland, CA, from January 2015 to February 2023 (*N* = 385)

	***N*/Median**	**%/IQR**
Demographics		
Age, year	58	54–62
Sex at birth		
Female	97	25.2
Transgender	4	1.0
Race		
Black	316	82.1
White	33	8.6
Latino/Hispanic	18	4.7
Mixed/other	18	4.7
Currently married or partnered	46	12.5
Years of education		
HS or more	282	74.0
Years homeless	3	1–10
Nights slept on street or in emergency shelter, past 6 months		
0 nights	121	31.4
1–150 nights	113	29.4
> 150 nights	151	39.2
Current housing situation		
Homeless	161	41.8
Not homeless	221	57.4
Lives in a skilled nursing facility	3	0.8
Received a housing voucher, subsidy, or PSH, past 6 months	14	3.6
Number of close confidants		
0	98	25.7
1–5	252	66.0
6 +	32	8.4
UCLA 3-item Loneliness Scale score	5	3–6
≥ 6	151	39.2
PROMIS Instrumental Support Scale score	16	11–30
Experienced hallucinations, 6 Mo	40	10.5
CESD score ≥ 22	120	32.1
Experienced anxiety, 6 Mo	137	35.7
PTSD score ≥ 3	94	24.4
Experienced suicidal ideation, 6 Mo	18	4.7
Experienced verbal abuse, 6 Mo	133	34.6
Experienced physical or sexual abuse, 6 Mo	63	16.4
Experienced verbal, physical, or sexual abuse as a minor	217	56.4
Experienced cognitive difficulty, 6 Mo	139	36.2
Any ADL difficulty	148	38.5
Experienced urinary incontinence, past month	161	42.4
Have trouble seeing, even w/glasses if needed	202	52.6
Have hearing problems	126	32.9
Experienced pain or took meds, past week	234	61.1
Experienced falls, 6 Mo	114	29.6
Number of chronic diseases	1	1—2
Has 2 or more chronic diseases	170	44.2
WHO ASSIST ≥ 4, cocaine	99	25.8
WHO ASSIST ≥ 4, amphetamines	25	6.5
WHO ASSIST ≥ 4, opiates	17	4.4
How often have a drink containing alcohol, past month		
Never	163	42.3
Once a week or less	101	26.2
2 + times per week	121	31.4
Six + drinks monthly or more frequently	42	11.0

**Figure 3:**
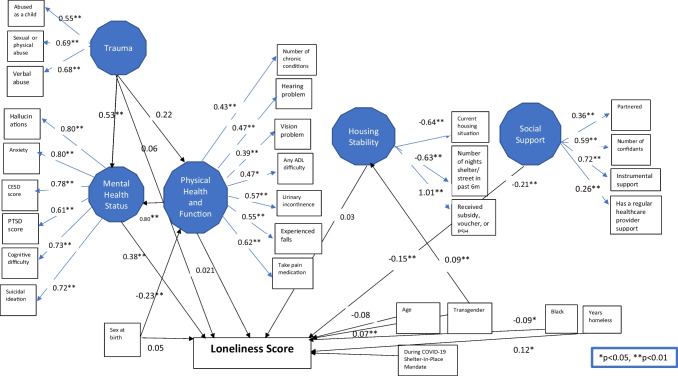
Final model (standardized coefficients, N = 385 respondents, 2099 visits)

The structural equation model identified key psychosocial and health factors associated with loneliness (Table 2; Fig. 3). Lower levels of social support were significantly associated with increased loneliness. Housing stability was not significantly associated with loneliness. Poorer mental health status was significantly associated with greater loneliness.

**Table 2:** Unstandardized Regression Weights (*B*) and 95% Confidence Intervals from the Final Structural Equation Model^a^

**Outcome **		**Explanatory**	**B**		**95% CI**		***p*-value**
Received housing voucher, subsidy, or PSH, 6Mo		Housing Stability	1.00		1.00, 1.00		
Nights Slept on Street or In Emergency Shelter, 6Mo		Housing Stability	−0.270		−0.38, −0.16		**
Current housing situation		Housing Stability	−0.63		−1.07, −0.19		**
Transgender		Housing Stability	0.81		0.45, 1.17		**
Number of Close Confidants		Social Support	1.00		1.00, 1.00		
Currently Married or partnered		Social Support	0.61		0.16, 1.07		**
PROMIS Instrumental Support Scale Score		Social Support	0.39		0.25, 0.54		**
Has a regular healthcare provider		Social Support	0.44		0.11, 0.78		**
Experienced Hallucinations, 6Mo		Mental Health	1.00		1.00, 1.00		
Experienced Anxiety, 6Mo		Mental Health	1.00		0.87, 1.14		**
CESD Score		Mental Health	0.19		0.16, 0.21		**
PTSD Score		Mental Health	0.27		0.21, 0.32		**
Experienced Suicide Ideation, 6Mo		Mental Health	0.90		0.76, 1.05		**
Experienced Cognitive Difficulty, 6Mo		Mental Health	0.92		0.79, 1.04		**
Any ADL Difficulty		Physical Health and Functional Status	1.00		1.00, 1.00		
Urinary Incontinence, Past Month		Physical Health and Functional Status	0.81		0.66, 0.96		**
Have Trouble Seeing, Even W/Glasses If Needed		Physical Health and Functional Status	0.55		0.33, 0.78		**
Hearing Problems		Physical Health and Functional Status	0.67		0.44, 0.89		**
Experience Pain or Took Meds, Past Week		Physical Health and Functional Status	0.88		0.75, 1.01		**
Ever Experienced Falls, 6 Mos		Physical Health and Functional Status	0.77		0.56, 0.99		**
Number of Chronic Diseases		Physical Health and Functional Status	0.83		0.58, 1.08		**
Sex at Birth		Physical Health and Functional Status	−0.37		−0.58, −0.15		**
Mental Health		Physical Health and Functional Status	0.74		0.17, 1.09		**
Experienced verbal abuse, 6Mo		Experience of Trauma	1.00		1.00, 1.00		
Experienced physical or sexual abuse, 6Mo		Experience of Trauma	1.01		0.84, 1.19		**
Experienced physical, sexual, or verbal abuse or inadequate food/shelter as a minor		Experience of Trauma	0.80		0.56, 1.05		**
Mental Health		Experience of Trauma	0.71		0.16, 1.14		*
Physical Health and Functional Status		Experience of Trauma	0.01		−0.17, 0.05		
Physical Health and Functional Status and Mental Health		Experience of Trauma	0.15		−0.11, 0.58		
UCLA 3-item Loneliness Scale Score		Housing Stability	0.05		−0.11, 0.21		
UCLA 3-item Loneliness Scale Score		Social Support	−0.49		−0.93, −0.04		*
UCLA 3-item Loneliness Scale Score		Mental Health	0.88		0.51, 1.26		**
UCLA 3-item Loneliness Scale Score		Experience of Trauma	0.17		−0.21, 0.55		
UCLA 3-item Loneliness Scale Score		Physical Health and Functional Status	0.06		−0.32, 0.45		
UCLA 3-item Loneliness Scale Score		Sex at birth	0.21		−0.09, 0.52		
UCLA 3-item Loneliness Scale Score		Transgender	1.12		0.46, 1.79		**
UCLA 3-item Loneliness Scale Score		Years homeless	1.05		0.31, 1.79		**
UCLA 3-item Loneliness Scale Score		Age	−0.72		−1.50, 0.07		
UCLA 3-item Loneliness Scale Score		Black	−0.46		−0.85, −0.07		*
UCLA 3-item Loneliness Scale Score		During COVID-19 SIP mandate	−0.31		−0.50, −0.12		**

*N *= 385 respondents, 2099 visits

* *p*<0.05, ** *p*<0.01

Physical health and functional status were indirectly associated with loneliness through mental health. The direct effect of physical health on loneliness was not significant, but the indirect effect via mental health was. The total effect was statistically significant, suggesting that worsening physical health and functional status was associated with increased loneliness via its negative impact on mental health status.

Trauma was indirectly associated with loneliness through mental health. While the direct path between trauma and loneliness was not significant, the indirect effect of the experience of trauma on loneliness via mental health was. The indirect effect of the experience of trauma on loneliness via physical health and functional status was not significant. The indirect effect of experience of trauma on loneliness via physical health and functional status and mental health was not significant. The total effect was significant, indicating that the experience of trauma was associated with increased loneliness via its negative impact on mental health status.

There was a statistically significant relationship between the SIP time period and reduced loneliness.

We included model fit statistics in the Appendix.

## DISCUSSION

In a longitudinal cohort study of older adults who were experiencing homelessness at study enrollment, we found a direct relationship between poor mental health and loneliness. Poor mental health was associated directly with loneliness and was linked to other factors associated with loneliness, such as poor physical health and trauma. We did not find an association between regaining housing and a decrease in loneliness, suggesting that providing housing alone may not be sufficient to alleviate loneliness in older homeless adults. These findings underscore the complex ways that mental health, physical health, trauma, and social support act to perpetuate or decrease loneliness.

Our results reinforce prior research indicating that poor mental health is a key driver of loneliness among older adults.^4,52,53^ Depression, anxiety, and psychological distress can contribute to social withdrawal, making it more difficult to maintain meaningful connections.^54^. Moreover, we found that physical health and functional impairments indirectly contributed to loneliness by worsening mental health, highlighting the interplay between physical and psychological well-being. Older adults with chronic illnesses or mobility limitations experience increased mental health distress, which in turn exacerbates loneliness.^55^ Addressing mental health concerns, alongside physical health, may be essential for mitigating loneliness in this population.

Consistent with existing literature,^6,8–11^ we found that a lack of social support was strongly associated with increased loneliness. Older adults who have experienced homelessness may have strained family ties or lost connections due to long-term housing instability,^56^ others may intentionally distance themselves from relationships that they perceive as harmful or unsupportive.^57^ Social support may be inconsistent or transactional—such as relying on acquaintances for temporary assistance—without the deeper emotional bonds needed to reduce loneliness.^57^ Structural barriers, including stigma, can further limit opportunities for rebuilding supportive relationships.^58–60^ Addressing loneliness in this population requires not only facilitating social connections but also fostering trust, stability, and reciprocity in relationships.

Experiences of trauma indirectly contributed to increased loneliness through their impact on mental health, highlighting the lasting psychological consequences of adversity. Many older adults who have experienced homelessness have endured multiple forms of trauma, including childhood adversity and interpersonal violence while experiencing homelessness.^57,61^ Trauma can shape perceptions of trust and safety, making it difficult to form and maintain close relationships, even when opportunities for social connection exist. Unaddressed trauma can contribute to depression and PTSD, which may exacerbate feelings of isolation.^62^ The cyclical nature of trauma, mental health struggles, and loneliness suggests that interventions should go beyond addressing immediate social needs to incorporating trauma-informed care to help individuals develop coping strategies, rebuild trust, and feel safe in relationships.

This study is among the first to investigate factors associated with loneliness among older adults who have experienced homelessness. Contrary to our hypothesis, we did not find an association between regaining housing and loneliness, suggesting that regaining housing itself does not alleviate loneliness. This finding aligns with emerging research showing mixed outcomes on social integration and loneliness among individuals moving from homelessness into permanent housing^63,64^ Individuals exiting homelessness may have difficulty adjusting to living alone after leaving the community they formed in shelters or homeless encampments. Others may face “survivor’s guilt,” feeling as though they have betrayed their social network if they gained housing and others did not.^65^ Others, in search of affordability or the ability to use rental subsidies, may need to leave the neighborhoods where they had community, fraying their social ties.^66^ Finally, stringent rules in some project-based PSH, such as not allowing guests to visit, may increase loneliness.^67^ These findings suggest that housing programs should incorporate strategies to support social integration.

Our findings indicate that loneliness decreased during the COVID-19 shelter-in-place (SIP) mandate. While this contrasts with research documenting increased loneliness among the general older population during this period,^67–69^ it aligns with research showing diverse experiences with SIP among vulnerable populations.^70^ Several contextual factors may help explain this unexpected trend. Targeted service adaptations, such as SIP hotel programs that temporarily housed unsheltered older adults, along with increased case management, wellness checks, and expanded tele-outreach, may have buffered the isolating effects of the pandemic.^71–73^ The structured nature of the SIP mandate may have alleviated certain daily stressors, such as navigating complex systems for basic needs. These findings underscore the context-dependent nature of loneliness and point to the need for future research to examine how sudden policy shifts and social disruptions shape loneliness trajectories among unstably housed populations.

Our findings suggest several key areas for intervention. First, integrated care models that address both mental and physical health may be essential in reducing loneliness among older adults with experiences of homelessness. Ensuring access to mental health services, particularly trauma-informed care, may help address underlying psychological distress that contributes to loneliness. Second, fostering social support through peer networks, community-based programs, and family reunification efforts may be crucial for addressing loneliness beyond housing stability. Finally, housing programs should prioritize social integration by creating opportunities for residents to build meaningful connections and engage in community life. To date, few interventions have been designed or evaluated to address loneliness among older adults with lived experience of homelessness. While many project-based Permanent Supportive Housing and senior housing facilities include communal spaces intended to foster social connection, their impact on loneliness has not been systematically evaluated, highlighting a critical gap for future program development and research.

Our study had several limitations. We did not include measures of the length of time in housing for those who were housed, which may have influenced loneliness levels. We did not account for housing tenure, housing type (e.g., own apartment versus staying with others; staying in a building with community space versus not), or neighborhood context, all of which may serve as potential confounders in the relationship between housing and loneliness. Our model does not account for the temporality or longitudinal relationships among variables, which limits our ability to assess how changes over time influence loneliness; future research should build on these findings to explore these dynamic. Due to small sample sizes, we collapsed race into a Black vs. non-Black, which may obscure meaningful heterogeneity in lived experiences, particularly related to nativity, immigration history, and patterns of segregation. Future research should prioritize larger and more diverse samples to allow for more nuanced analyses of racialized experiences.

## CONCLUSIONS

Loneliness is a significant concern for older adults with experiences of homelessness, influenced by mental health, physical health, trauma, and social support. Our findings underscore the need for comprehensive, trauma-informed interventions that address both psychological and social aspects of loneliness. Future policy should integrate mental healthcare, foster social connections, and create supportive housing environments, with further research on the long-term impacts of these approaches.

## APPENDIX

Model fit statistics: In the initial model (Fig. 1), we had a satisfactory model fit (*χ*2[503] = 480.71, *p* = 0.76; CFI = 1.000; SRMR = 0.116; RMSEA = < 0.000). Removing an insignificant path (*p* > 0.05) between substance abuse and loneliness did not affect model-data fit and met our a priori criteria. The chi-square fit index for the revised model was *χ*2[375] = 355.64, *p* = 0.76; CFI = 1.000; SRMR = 0.136; and RMSEA = < 0.000.^74^

## Data Availability

The datasets generated and analyzed during the current study are not publicly available but are available from the corresponding author on reasonable request.
